# A new human opisthorchiasis outbreak in central Italy: a never-ending story

**DOI:** 10.1007/s15010-024-02340-8

**Published:** 2024-09-26

**Authors:** Chiara Papalini, Maria Angeles Gómez-Morales, Alessandra Mercuri, Elisa Stolaj, Maria Grazia Brancaleoni, Igino Fusco Moffa, Giovanni Lo Vaglio, Alessandra Ludovisi, Gianluca Marucci, Daniela Francisci

**Affiliations:** 1 Infectious Diseases Clinic, Santa Maria della Misericordia Hospital, University of Perugia, Piazzale Gambuli 1, Perugia, 06100 Italy; 2 Department of Infectious Diseases, European Union Reference Laboratory for Parasites, Istituto Superiore di Sanità, Roma, Italy; 3 Department of Prevention, Travel Medicine Unit, Local Health Unit Umbria 1, Perugia, Italy; 4 Department of Prevention, Hygiene of food of animal origin, Local Health Unit Umbria 1, Trasimeno area, Panicale, Perugia, Italy

**Keywords:** Opisthorchis, Zoonosis, Trematode, Cholestasis, Eosinophilia, Praziquantel

## Abstract

**Purpose:**

*Opisthorchis felineus* is a trematode causing a foodborne infection transmitted by raw freshwater fish belonging to Cyprinidae family. Human outbreaks in Italy dated back to 2003–2011 and involved lakes of Central Italy. The aim of this study is to report epidemiological and clinical characteristics of the human opisthorchiasis outbreak occurred in Central Italy in 2022 comparing it with previous events.

**Methods:**

We report cases diagnosed from June to December 2022 in Perugia hospital thanks to serological and molecular tests and direct examination of feces.

**Results:**

Sixty-seven individuals were traced back by epidemiological investigation. Forty-seven received a diagnosis of opisthorchiasis, of which 45 were confirmed cases and two were considered as probable cases. These 47 individuals attended a Trasimeno lakeshore restaurant in May 2022. All but 20 presented symptoms, mostly fever. Sixteen (15 confirmed and 1 probable) cases required hospitalization. Feces examination revealed *Opisthorchis* spp. eggs in 35/45 (78%) confirmed cases. Thirty individuals underwent to serology and molecular stool test: 5 (16.7%) results positive to the former, 1 (3.3%) to the latter while 4 (13.3%) to both. Laboratory tests, available in 28 patients, showed eosinophilia in 82.1%, increase of alanine aminotransferase, gamma-glutamyl transferase and alkaline phosphatase in 64.3%, 75% and 67.9%, respectively. Because of pharmacy shortage of praziquantel, 22 patients were treated with albendazole, of which 13 failed clearing the parasite.

**Conclusion:**

Opisthorchiasis still represents a challenging diagnosis, in particular for asymptomatic patients. Albendazole may lead to treatment failure. Control measures in known endemic areas should be implemented.

**Trial registration:**

number 27,498/23/ON, approved by Ethical Committee of Umbrian Region in 09.13.2023.

## Background

Opisthorchiasis is a zoonosis caused by trematodes of the genus *Opisthorchis* through the ingestion of raw or partially cooked freshwater infected fish belonging to the Cyprinidae family. The adult worm’s habitat are intra-hepatic biliary ducts of fish-eating mammals (mainly felids and canids) while humans are occasional definitive hosts. *Opisthorchis* spp. eggs shed with feces can contaminate fresh water lakes and rivers. Fresh-water snails of genus *Bithynia* (first intermediate host) can ingest the eggs. Inside the snail, the miracidium comes out the egg and develops to a sporocyst, then to redia and finally to cercaria, which actively emerge from the mollusk mantle. Cercariae leave the snail during the day, swim in search of fish (second intermediate host), penetrating between the scales and, finally, in muscles they develop to metacercaria, the infective form. Of the two species of the genus *Opisthorchis, Opisthorchis felineus* is present in Asia and Europe and *Opisthorchis viverrini* in South Eastern Asia. *O. felineus* infects 1.2 million people in Eastern Europe and Russia [1] and some hundreds of cases have been reported in the European Union [2]. In Central Italy lakes of volcanic origin namely Bolsena, Bracciano and Vico (Latium region), *O. felineus* is present [3] and outbreaks took place for the consumption of tenches fished in these lakes from 2003 to 2011 [2]. However, is under debate whether the two opisthorchiasis outbreaks occurred in 2003 and 2006 in Trasimeno Lake (Umbria region) were autochthonous or imported from endemic lakes in neighboring Latium region [2, 4, 5].

As other liver flukes, the magnitude of the pathology caused by *O. felineus* depends on the parasitic burden, the susceptibility of the host and the occurrence over the time of infections/reinfections causing ongoing inflammatory conditions. The intraductal flukes move up to and clog biliary branches causing a mechanical injury by their feeding and migrating activities, both oral and ventral suckers of the fluke hook up the biliary epithelium. Moreover, the excretory/secretory products released by the parasites induce inflammation and stimulate cell proliferation of the biliary epithelium, causing immunopathological responses [6]. *O. felineus* chronic infections may cause complications, such as cholangitis, cholecystitis, cholangiofibrosis, hepatic cysts, hepatic abscesses and pancreatitis [7]. While *O. viverrini* (and *Clonorchis sinensis*) infections were associated with cholangiocarcinoma (IARC Group 1: carcinogenic), so far such an association has not been reported for *O. felineus* and cholangiocarcinoma (IARC Group 3: unclassified) [8, 9]. However, epidemiological and clinical data in humans and animals suggest that *O. felineus* can be the cause of neoplasia in chronic infections [2, 8–13].

The aim of this study is to report epidemiological and clinical characteristics of the last opisthorchiasis outbreak occurred in May 2022 in Umbria region (Central Italy), and to compare them with previous Italian episodes. The recurrence of these events highlights the need of implementing control measures in known endemic areas. This is particularly important as there might be asymptomatic and undiagnosed non-treated cases during the acute phase that may tend to chronicity, as elicited in this study and elsewhere [14].

## Materials and methods

This prospective cohort study involved symptomatic and not symptomatic patients, who ate marinated filets in the restaurant of Sant’Arcangelo (Magione, Perugia, Italy) in May 2022 and were diagnosed with an *O. felineus* infection from June to December 2022. The identification of the etiological agent was based first on the morphology of the eggs by microscopic examination of three samples of formol-ether concentrated feces collected at three different days per patient, and then the identification was confirmed by PCR [15]. This highly specific assay is able to amplify the ITS2 region of the rDNA of the parasite eggs and metacercariae, providing a 250-bp fragment that can be sequenced and compared with the internal transcribed spacer (ITS2) sequences of *O. felineus*, *O. viverrini*, and *Clonorchis sinensis* present in the GenBank database. Patients resulted negative underwent a serological test to detect anti-*Opisthorchis* IgG, and a conventional PCR in faecal samples for detection of Opisthorchiidae DNA as above [15, 16].

Confirmed cases were those resulting positive by a test in feces for *O. felineus* (eggs or DNA) and/or for anti-*Opisthorchis* IgG in serum, and an epidemiological link to the cluster [14]. Probable cases were those having clinical manifestations compatible with opisthorchiasis and an epidemiological link to the cluster.

During the visit, the following epidemiological and clinical data were collected: patients’ age and gender, signs, symptoms, eaten food, date of restaurant dinner or lunch, date of symptoms onset and their duration, hospitalization (if any), ultrasonography findings when performed, date and treatment carried out, blood tests (blood counts, bilirubin, C-reactive protein (C-RP), alanine aminotransferase (ALT), aspartate aminotransferase (AST), gamma-glutamyltransferase (GGT), alkalin phosphatase (ALP)) before and one month after therapy. Clinical conditions and parasitological tests at one, two, six and 12 months follow up were also recorded, as results of DNA assay on stool and serology at 3 and 12 months.

Continuous variables are presented as the median with interquartile range (IQR), while the categorical ones were expressed by their relative frequencies and ranges. A comparison of treatment efficacy was also performed between patients treated with praziquantel (PZQ) and albendazole (ABZ). In the ABZ group, a comparison between cases with therapeutic success or failure was also accomplished. The t Student test, Mann-Whitney U-test and the Fisher’s exact test were used to compare differences between groups, as appropriate. A *p* value < 0.05 was considered significant.

## Results

In June 2022, 11 index patients with fever and/or abdominal disturbance were admitted at Infectious Diseases Clinic of Santa Maria della Misericordia Hospital (Perugia University, Umbria region, Italy). The anamnestic investigation revealed that all of them eat a meal at a lakeshore restaurant in Sant’Arcangelo (Magione, Perugia, Italy) on 15th, 19th and 21st May 2022. In the light of these findings, epidemiological analysis were focused on customers of that restaurant in those days. In December 2022, another patient complaining hepatic problems came to Infectious Diseases Clinic’s attention. He was a professor and had visited the same restaurant on May 20th 2022 with his students who were subsequently involved in contact tracing.

Overall, the epidemiological investigation traced back 67 exposed people, of them 45 opisthorchiasis confirmed cases (attack rate 67.2%) and 2 probable cases, living in Lombardy region, who were referred to local hospital and were treated with PZQ only based on clinical features, without performing any specific diagnostic test for *Opisthorchis* infection. Among these 47 cases, 22 (46.8%) males, mean age 45.3 years, 41 (87.2%) were Umbria region inhabitants while the remaining from Tuscany (1), Latium (3) and Lombardy regions (2). Patients remembered having eaten marinated filets of Largemouth bass (*Micropterus salmoides*) or common whitefish (*Coregonus* sp.) and tench pate (*Tinca tinca*) as starter.

Considering just confirmed diagnoses, symptomatic individuals were 25 (55.5%) and 15 (33.3%) of them required hospitalization due to fever and/or abdominal pain. The median of the signs and symptoms onset was 16.5 days (IQR 13.75–21.25) after fish consumption while definitive diagnosis took a median of 14 (IQR 7-22.5) days between signs and symptoms onset and positive parasite examination of stool. Clinical and laboratory features of the cases are presented in Table 1.

**Table 1:** Clinical and laboratory features of opisthorchiasis cases at the admission as inpatients or outpatients

Clinical features in confirmed + probable cases (*N* = 45 + 2)	*N* (%)
Males	21 + 1 (46.8)
Hospitalized	15 + 1 (34)
Symptomatic individuals Fever > 38 °C	25 + 2 (57.4) 19 + 2 (44.7)
Abdominal discomfort	13 + 2 (31.9)
Asthenia	7 + 1 (47)
Myalgia	7 (14.9)
Diarrhoea	5 (10.6)
Vomiting	4 (8.5)
Rash	4 (8.5)
Sine materia pruritus	3 (6.3)
**Parasitological tests** (*n* = 45)^a^	
*Opisthochis* eggs in faeces	35 (78)
*Opisthorchis felineus* DNA in faeces	5 (11.1)
anti-*Opisthorchis* IgG in serum	9 (20)
**Laboratory features** (*n* = 28^b^)	N (%); range
Eosinophilia^c^	23 (82.2); (239 − 23,765 cells/µl)
Increased levels of GGT	21 (75); (17–876 UI/L)
Increased levels of ALP	19 (67.9); (58–999 UI/L)
Increased levels of ALT	18 (64.2); (19–457 UI/L)
Increased levels of AST	12 (42.9); (16–183 UI/L)
Increased levels of bilirubin	8 (28.6); (0.31–2.61 mg/dl)
Increased levels of C-RP	10 (35.7); (0.05–10.6 mg/dl)

^a^ Two individuals living in Lombardy referred to local hospital and were treated based on clinical features, without performing any diagnostic test

^b^ Twenty five symptomatic and three asymptomatic patients

^c^ Higher values than: 500 cells/µl (eosinophils); 50 UI/L (ALT); 50 UI/L (AST); 55 UI/L (GGT); 120 UI/L (ALP); 1.2 mg/dl (Bilirubin); 0.5 mg/dl (C-RP)

Microscopic examination of feces resulted positive for *Opisthorchis* spp. eggs in 35 (77.8%) patients. Fecal samples of asymptomatic persons with negative results were tested by PCR and underwent a serologic test to detect anti-*Opisthorchis* IgG. Four (8.9%) individuals resulted positive by both tests, five (11.1%) positive only by serology and one only by PCR as she did not authorized any blood sampling. (Table 1).

Blood tests were obtained from 28 individuals, 25 of them were symptomatic and showed increase of several parameters (Table 1). Ultrasonography performed in 17 (37.8%) patients showed gallbladder wall thickening in 13 (76.5%) individuals. Except for an asymptomatic 74-year-old patient with cirrhosis due to non-infective causes, all the confirmed cases were treated: 22 (48.9%) with PZQ (25 mg/kg orally thrice a day for 2 days treatment) and 22 (48.9%) with ABZ (10 mg/kg/day, twice per day, for 7 days treatment).

Of the 22 patients treated with ABZ, 13 still had *Opisthorchis* eggs in stool at one (*n* = 8, 36.4%), two (*n* = 4, 18.2%) and six (*n* = 1, 4.5%) months post treatment. Seven (53.8%) were males, with a mean age of 52 years old. Out of these 13 patients, 6 (46.1%) had pathologic ultrasonography findings, 8 (61.5%) increased ALT and 10 (76.9%) eosinophilia and hyper-GGT at the diagnosis. Twelve (92.3%) out 13 individuals failing ABZ therapy, were symptomatic at diagnosis and 7 (53.8%) also after treatment. Therefore, all of them received a second treatment with PZQ.

No therapeutic failure was noticed in the 22 confirmed cases firstly treated with PZQ neither in the 13 individuals treated with this drug after the treatment with ABZ. Laboratory follow up at one-month after treatment was available for 12 patients treated with ABZ and 7 treated with PZQ as first anti-parasite therapy. No statistically significant differences were found in laboratory parameters between the two treatments (Table 2).

**Table 2:** Laboratory features of patients treated with ABZ and PZQ one month after the treatment

	After therapy			
		ABZ (*N* = 12) N (%)	PZQ (*N* = 7) N (%)	*P*
Eosinophilia^a^		7 (58.3)	2 (28.6)	0.3498
Increased levels of ALT		4 (33.3)	1 (14.3)	0.6027
Increased levels of AST		0	1 (14.3)	0.3684
Increased levels of GGT		7 (58.3)	5 (71.4)	0.6562
Increased levels of ALP		3 (25)	3 (42.8)	0.6169
Increased levels of bilirubin		0	0	1

^a^ Higher values than: 500 cells/µl (eosinophils); 50 UI/L (ALT); 50 UI/L (AST); 55 UI/L (GGT); 120 UI/L (ALP); 1.2 mg/dl (Bilirubin)

Nine patients with a previous positive serology or molecular stool assay, repeated the same tests three and 12 months after therapy. Even with lower values than in the previous checks, positive serology persisted in six (at 3 months p.i.) and three (at 12 months p.i.) patients, respectively, while the PCR for *O. felineus* on feces tested negative in all the cases (Table 3).

**Table 3:** Follow-up of patients, at definitive diagnosis and at different time points after treatment

	At diagnosis *N* (%)			Follow up *N* (%)	
		1 month N (%)	2-3^a^ months N (%)	6 months N (%)	1 year N (%)
*Opisthorchis* sp. eggs in stool	35 (77.8)	8/26^b^ (30.8)	4/16^b^ (25)	1/21^b^ (4.8)	0/22^b^
anti-*O. felineus* IgG	9^c^ (30)	-	6/9 (66.7)	-	3/9 (33.3)
*O. felineus* DNA in stool	5^c^ (16.7)	-	0/5 (0)	-	-
Eosinophilia	23/28 (82.1)	10/28 (35.7)	1/28 (3.6)	0/28 (0)	-
Hyper-GGT	21/28 (75)	12/28 (42.9)	4/28 (14.3)	1/28 (3.6)	-
Hyper-ALT	18/28 (64.3)	5/28 (17.9)	2/28 (7.1)	0/28 (0)	-

^a^2 months for stool direct examination and 3 months for serology and DNA assay on stool

^b^The individuals lacking to reach 35 missed the corresponding follow up

^c^Thirty patients tested for serology and molecular stool test

Of the 67 exposed people, 20 asymptomatic individuals which had resulted negative for opisthorchiid eggs in feces, tested negative for parasitic DNA in stool and anti-*O. felineus* IgG in serum at five-eight months after exposure.

## Discussion

Infections caused by *O. felineus* have been periodically reported and originated from Central Italy lakes (Table 4). The prevalence of *O. felineus* in tenches varies from 0% in Trasimeno Lake to 74% and 95% in Bolsena and Bracciano lakes, respectively [2, 3]. In humans, from 2003 to 2022, a total of 259 individuals have been reported to be infected with this parasite, all living or travelling to Latium or Umbria regions (Central Italy) [14, 17–19, present study]. An exception is represented by an outbreak in Aosta Valley region (Northern Italy) in 2010, where people was infected by tenches fished in the Bracciano lake (Latium region) and marketed in northern Italy [17, 18], (Table 4). Epicenter of the present outbreak was a restaurant managed by the fishermen cooperative owning the annex fish market, too. Case tracing was extremely difficult, therefore the real number of exposed people might have been underestimated due to the absence of a customers’ list. The outbreak, consequently, could have spread with tourists, who became infected at the restaurant and could have developed the disease upon returning home, as occurred in the past [18–20]. Epidemiological inquiry started after hospitalized patients referred to have had a meal at the same restaurant on precise dates (15th, 19th, 20th and 21st May 2022), which led to trace their diners. The attention was focused on the starters because the majority of patients remembered eating marinated filets of Largemouth bass or *Coregonus* sp. and smoked tench pate. Unfortunately, no fish sample resulted positive for *Opisthorchis* spp. metacercariae, even if the tench remained the major suspect. However, Gustinelli’s report on Trasimeno infestation revealed scarcity of suitable intermediate hosts (*Bithynia*) for *O. felineus*, irrespective the high *Opisthorchis* prevalence in cats’ feces [21]. It is possible that fishes responsible of Trasimeno’s outbreaks were imported from Bolsena Lake, or that among the filets offered as white fish (*Coregonus* sp.), there were some infected tenches originated from other central Italian lakes, which are less expensive than the white fish [3], as happened in the past [14, 22].

**Table 4:** Epidemiological and clinical characteristics of Italian outbreaks of opisthorchiasis

	2003	2005	2007	2008	2009	2010	2011	2022	
Outbreak epicenter	Trasimeno Lake	Trasimeno Lake	Bolsena Lake	Bolsena Lake	Bolsena Lake	Aosta Valley	Bolsena Lake	Trasimeno Lake	
Fish origin	Bolsena Lake	Bolsena Lake	Bolsena Lake	Bolsena Lake	Bolsena Lake	Bracciano Lake	Bolsena Lake	Bolsena Lake?	
Place of fish consumption	restaurant	restaurant	restaurant	home	catering	restaurant	restaurant	restaurant	
Exposition-onset interval (days)	27	60	14–30	NA	14–30	7–37	28	0–87	
Onset-diagnosis interval (days)	30	NA	NA	NA	60–120	NA	NA	3-160	
**Number/total tested (%)**									Total
Individuals involved	2	8	34	NA	44	93	500	67	748
Affected patients	2/2 (100)	8/8 (100)	22/34 (64.7)	4	31/44 (70.4)	60/93 (64.5)	80/100	47/67 (70.1)	254
Male gender	1 (50)	5 (62.5)	NA	NA	23 (74.2)	NA	NA	22 (46.8)	51
Mean age (years)	42	29	NA	NA	44.9	NA	NA	45.3	NA
Hospitalized patients	1/2 (50)	1/8 (12.5)	2/22 (9.1)	2/4 (50)	0	13/60 (21.7)	19/80 (23.7)	16/47 (34)	54
Symptomatic patients	1/2 (50)	1/8 (12.5)	12/22 (54.5)	2/4 (50)	12/31 (38.7)	52/60 (86.7)	56/100 (56)	27/47 (57.4)	163
Fever	1/2 (50)	0	12/22 (54.5)	NA	12/31 (38.7)	29/45^a^ (64)	NA	21/47 (44.7)	75
Abdominal disturbance	0	1/8 (12.5)	12/22 (54.5)	NA	5/31 (16.1)	22/45^a^ (49)	NA	15/47 (31.9)	55
Asthenia	0	0	0	NA	12/31 (38.7)	14/45^a^ (31)	NA	8/47 (17)	34
Myalgia	0	0	12/22 (54.5)	NA	10/31 (32.2)	12/45^a^ (27)	NA	7/47 (14.9)	41
Eosinophilia	1/1^a^ (100)	1/3^a^ (33)	21/21^a^ (100)	NA	8/31 (26)	42/45^a^ (93)	NA	23/28 (82.1)	96
Increased ALT	NA	NA	9/21^a^ (42.9)	NA	4/31 (13)	NA	NA	18/28 (64.3)	31
Increased GGT	1^b^	NA	NA	NA	11/31 (35)	35/45^a^ (78)	NA	21/28 (75)	69
Treated with PZQ	0	NA	9/21^a^ (42.9)	NA	3/31 (9.7)	NA	NA	24/47 (51.1)	36
Treated with ABZ	0	NA	12/21^a^ (57.1)	NA	28/31 (90.3)	NA	NA	22/47 (46.8)	62
Retreated due to failure	0	NA	1/21 (4.8)	NA	1/31 (3.2)	NA	NA	13/47 (27.6)	15

NA: not available data; ^a^information about remnants unknown

In the present outbreak, symptomatic patients presented mainly fever and eosinophilia as already reported as the most frequent signs in human infections [14, 17, 23], (Table 4). The common presence of fever in a period still affected by COVID pandemic and the old age of some patients reporting other symptoms, as abdominal pain, were the leading causes for hospital admission. Liver enzymes were altered, however no case of jaundice was observed. Other non-specific signs and symptoms were present and laboratory data (bilirubin and C-RP) were increased with not such high frequency (Table 1), which could be the cause of the late diagnosis (median of 14 days). The diagnosis of opisthorchiasis is hampered by the lack of pathognomonic signs and symptoms and the decreasing number of health professionals with the skills needed to identify *Opisthorchis* spp. eggs in stool samples. On the other hand, the sensitivity of microscopy is low, in particular in the early disease stage, since egg production starts 1–3 months after infection [2, 19]. Furthermore, many infected persons show pauci-symptomatic or asymptomatic forms (as shown in Table 4) which can make diagnosis more complicated and lead to chronic infections [14]. In fact, from 2003 to 2011, in nine outbreaks and four single cases recorded in Italy, 72 (34.1%) out of 211 cases were asymptomatic [2]. Therefore, it is important that, in presence of epidemiological and clinical suspicion of opisthorchiasis, the diagnostic pathway combines parasitic stool examination with a serological test if available, since the time between infection and the detection of specific IgG in serum usually ranges from three to 8 weeks [2]. In the Italian outbreaks of opisthorchiasis including the present outbreak, the high number of asymptomatic patients suggests that persons ingested only once a low infecting dose. These epidemiological and clinical patterns are different from those observed in endemic foci of *O. felineus* in Eastern Europe and Western Siberia, where people frequently consume infected fish and clinical pictures are severe [7, 10, 12]. In this study, five asymptomatic cases that had tested negative for *Opisthorchis* eggs were detected only by serology. In a previous outbreak, of the 8 asymptomatic patients, three showed a positive serology with no eosinophilia or altered liver enzymes [17]. Moreover, serology resulted a good tool for the follow up of the patients after therapy, even if persisting anti-*O. felineus* IgG was detected in three patients one year p.i., its value slowly decreased after treatment (Table 3).

Twenty-two patients diagnosed with opisthorchiasis were treated with ABZ, considered less effective than PZQ, so a second-line drug [1, 14]. That was due to PZQ shortage in the hospital pharmacy and to its unavailability in Italy for the human use [7]. In this study, there was a conspicuous number of ABZ treatment failure, indeed, in addition to positive fecal examination, 7 (53.8%) of them still had symptoms at 1-month follow up after therapy. At the diagnosis, no significant differences about mean age, eosinophils, ALT and GGT basal values were noticed between patients successfully treated or not successfully treated by ABZ. One month after treatment, no statistical differences were found in terms of laboratory values between patients treated with ABZ or PZQ, however, the small sample size might constitute the main limitation of this analysis (Table 2).

In conclusion, in this outbreak epidemiological and clinical findings were similar to those of previous Italian events but with higher incidence of symptomatic and hospitalized patients. Unfortunately, also ABZ unsuccessful treatments were more frequent herein than in previous outbreaks, which were not associated to the persistence of cholestasis or eosinophilia, as happened in other cases (Table 4) [17, 23].

Following our report, local health authorities investigated restaurant and its fish and fish-based delicatessen. Until the day of publication, no further cases of opisthorchiasis were detected.

Opisthorchiasis is a continuous threat in endemic areas and climate change could enhance this fact as many studies indicate a positive effect of increasing temperature on lifespan, cercariae shedding and period of transmission of cercariae to the second intermediary host [24]. From a clinical point of view, the diagnosis may be difficult because it requires a careful anamnesis analysis of the previous 2–4 weeks. It is worth to consider this infection in differential diagnosis of eosinophilia and cholestatic hepatitis, especially in raw or partially cooked freshwater fish consumers who visited endemic areas. Moreover, laboratory parameters could be useful as alert signs also in asymptomatic individuals. Considering the amount of treatment failure with ABZ and the complications of the disease, PZQ supplying is crucial. To be successful and long-lasting, prevention programs need time, resources and community participation. Implementation of control measures in known endemic areas means improvements in hygiene to prevent transmission of eggs from feces and limiting snail population with molluscicides; however, the biggest case against this approach is that it is a huge interference in biocenosis that can have dramatic consequences [25]. The easiest and best way of preventing opisthorchiasis is decontaminating freshwater fish freezing it at -10 °C for 5–70 days or cooking until the fish core reaches 65 °C for at least 1 min [26].

## Data Availability

No datasets were generated or analysed during the current study.

## References

[1] WHO. (1995) Control of foodborne trematodes infection. WHO Technical Report Series 849. 7740791

[2] Pozio E, Armignacco O, Ferri F, Gómez-Morales MA. *Opisthorchis Felineus*, an emerging infection in Italy and its implication for the European Union. Acta Trop. 2013;126(1):54–62. 10.1016/j.actatropica.2013.01.005. 23337391 10.1016/j.actatropica.2013.01.005

[3] De Liberato C, Scaramozzino P, Brozzi A, Lorenzetti R, Di Cave D, Martini E, Lucangeli C, Pozio E, Berrilli F, Bossù T. Investigation on *Opisthorchis felineus* occurrence and life cycle in Italy. Vet Parasitol. 2011;177(1–2):67–71. 10.1016/j.vetpar.2010.11.042. 21168274 10.1016/j.vetpar.2010.11.042

[4] Crotti D, Crotti S. *Opisthorchis felineus* in deiezioni fecali della popolazione felina dell’Isola Maggiore Del Trasimeno (PG). Prog Vet. 2007;6:272–74.

[5] Crotti D, D’Annibale ML, Crotti S. Opistorchiasi Autoctona Del Lago Trasimeno (Perugia): descrizione di due Episodi Epidemici Da *Opisthorchis felineus* e problematiche diagnostiche differenziali. Microbiol Med. 2007;22(1):36–41. 10.4081/mm.2007.2896.

[6] Pozio E, Gómez-Morales MA. Clonorchiasis and Opisthorchiasis. In: Bruschi F, editor. Helminth infections and their impact on global public health. Springer. 2022:221–56.

[7] Pakharukova MY, Mordvinov VA. The liver fluke *Opisthorchis felineus*: biology, epidemiology and carcinogenic potential. Trans R Soc Trop Med Hyg. 2016;110:28–36. 10.1093/trstmh/trv085. 26740360 10.1093/trstmh/trv085

[8] Bouvard V, Baan R, Straif K, et al. A review of human carcinogens-part B: biological agents. Lancet Oncol. 2009;10:321–2. 10.1016/s1470-2045(09)70096-8. 19350698 10.1016/s1470-2045(09)70096-8

[9] Carcinogenic Hazards to Humans (IARC). List of classification. https://monographs.iarc.who.int/list-of-classifications/ [cited 2023 March 7].

[10] Mordvinov VA, Yurlova NI, Ogorodova LM, Katokhin AV. *Opisthorchis felineus* and *Metorchis bilis* are the main agents of liver fluke infection of humans in Russia. Parasitol Int. 2012;61:25–31. 10.1016/j.parint.2011.07.021. 21840415 10.1016/j.parint.2011.07.021

[11] Maksimova GA, Pakharukova MY, Kashina EV, Zhukova NA, Kovner AV, Lvova MN, et al. Effect of *Opisthorchis felineus* infection and dimethylnitrosamine administration on the induction of cholangiocarcinoma in Syrian hamsters. Parasitol Int. 2017;66:458–63. 10.1016/j.parint.2015.10.002. 26453019 10.1016/j.parint.2015.10.002 PMC4956575

[12] Kovner AV, Pakharukova MY, Maksimova GA, Mordvinov VA. Characteristics of liver fibrosis associated with chronic *Opisthorchis felineus* infection in Syrian hamsters and humans. Exp Mol Pathol. 2019;110:104274. 10.1016/j.yexmp.2019.104274. 31226265 10.1016/j.yexmp.2019.104274

[13] Lvova MN, Tangkawattana S, Balthaisong S, Katokhin AV, Mordvinov VA, Sripa B. Comparative histopathology of Opisthorchis Felineus and Opisthorchis viverrini in a hamster model: an implication of high pathogenicity of the European liver fluke. Parasitol Int. 2012;61:167–72. 10.1016/j.parint.2011.08.005. 21854870 10.1016/j.parint.2011.08.005

[14] Armignacco O, Ferri F, Gómez-Morales MA, Caterini L, Pozio E. Cryptic and asymptomatic *Opisthorchis felineus* infections. Am J Trop Med Hyg. 2013;88:364–66. 10.4269/ajtmh.2012.12-0280. 23249682 10.4269/ajtmh.2012.12-0280 PMC3583331

[15] Müller B, Schmidt J, Mehlhorn H. PCR diagnosis of infections with different species of Opisthorchiidae using a rapid clean-up procedure for stool samples and specific primers. Parasitol Res. 2007;100:905–9. 10.1007/s00436-006-0321-x. 17061114 10.1007/s00436-006-0321-x

[16] Gómez-Morales MA, Ludovisi A, Amati M, Pozio E. Validation of an Excretory/Secretory Antigen based-Elisa for the diagnosis of *Opisthorchis felineus* infection in humans from low Trematode endemic areas. PLoS ONE. 2013;8(5):e62267. 10.1371/journal.pone.0062267. 23671589 10.1371/journal.pone.0062267 PMC3650035

[17] Traverso A, Repetto E, Magnani S, Meloni T, Natrella M, Marchisio P, et al. A large outbreak of *Opisthorchis felineus* in Italy suggests that opisthorchiasis develops as a febrile eosinophilic syndrome with cholestasis rather than a hepatitis-like syndrome. Eur J Clin Microbiol Infect Dis. 2012;31(6):1089–93. 10.1007/s10096-011-1411-y. 21938537 10.1007/s10096-011-1411-y

[18] Vondeling AM, Lobatto S, Kortbeek LM, Naus H, Dorigo-Zetsma JW. Fever, malaise and eosinophilia after consumption of raw fish in Italy: infection by a liver fluke (*Opisthorchis Felineus*). Ned Tijdschr Geneeskd. 2012;156:A3873. 22296896

[19] Wunderink HF, Rozemijer W, Wever PC, Verweij JJ, van Lieshout L. Foodborne Trematodiasis and *Opisthorchis Felineus* Acquired in Italy. Emerg Infect Dis. 2014;20(1):154–55. 10.3201/eid2001.130476. 24520562 10.3201/eid2001.130476 PMC3884715

[20] Brugioni L, Tognetti M, Gozzi C. An unhealthy holiday on Lake Bolsena. Italian J Med. 2013;7(1):39–42. 10.4081/itjm.2013.39.

[21] Gustinelli A. Elminti di interesse zoonosico in specie ittiche dulciacquicole nazionali. Tesi di Dottorato in Epidemiologia e Controllo delle Zoonosi (ciclo XX), settore VET-06, Alma Mater Studiorum Bologna, http://amsdottorato.unibo.it/816/2/Gustinelli_Tesi_Dottorato_Epidemiologia_e_controllo_delle_zoonosi__XX_ciclo.pdf.

[22] Cacciò SM, Chalmers RM, Dorny P, Robertson LJ. Foodborne parasites: outbreaks and outbreak investigations. A meeting report from the European network for foodborne parasites (Euro-FBP). Food Waterborne Parasitol. 2018;8(10):1–5. 10.1016/j.fawpar.2018.01.001. 10.1016/j.fawpar.2018.01.001 PMC7033997 32095595

[23] Armignacco O, Caterini L, Marucci G, Ferri F, Bernardini G, Natalini Raponi G, et al. Human illnesses caused by *Opisthorchis felineus* flukes, Italy. Emerg Infect Dis. 2008;14(12):1902–5. 10.3201/eid1412.080782. 19046516 10.3201/eid1412.080782 PMC2634636

[24] Ponomareva NM, Orlova TV, Vlasenko PG, Serbina EA, Yurlova NI. Temperature dependence of *Opisthorchis felineus* infection in the first intermediate host snail, *Bithynia Troschelii*. Acta Trop. 2024;253:107166. 10.1016/j.actatropica.2024.107166. 38431135 10.1016/j.actatropica.2024.107166

[25] Sithithaworn P, Andrews RH, Mordvinov VA, Pakharukova MY, Lvova MN. *Opisthorchis viverrini* and *Opisthorchis Felineus*. Encyclopedia of Food Safety. Academic; 2024. pp. 673–68.

[26] European Food Safety Authority. Scientific opinion on risk assessment of parasites in fishery products. EFSA J. 2010;8:1543. 10.2903/j.efsa.2010.1543.

